# Chemotactic migration of newly excysted juvenile *Clonorchis sinensis* is suppressed by neuro-antagonists

**DOI:** 10.1371/journal.pntd.0007573

**Published:** 2019-08-13

**Authors:** Shunyu Li, Jin-Ho Song, Tae Im Kim, Won Gi Yoo, Moo-Ho Won, Fuhong Dai, Sung-Jong Hong

**Affiliations:** 1 Department of Medical Environmental Biology, Chung-Ang University College of Medicine, Seoul, Republic of Korea; 2 Department of Pharmacology, Chung-Ang University College of Medicine, Seoul, Republic of Korea; 3 Division of Planning and Management, Nakdong-gang National Institute of Biological Resources, Sangju, Gyeongsangbuk-do, Republic of Korea; 4 Department of Neurobiology, School of Medicine, Kangwon National University, Chuncheon, Gangwon-do, Republic of Korea; 5 Department of Parasitology, Medical College of Soochow University, Suzhou Industrial Park, Suzhou, Jiangsu, P.R. China; Seoul National University College of Medicine, REPUBLIC OF KOREA

## Abstract

The metacercariae of the *Clonorchis sinensis* liver fluke excyst in the duodenum of mammalian hosts, and the newly excysted juveniles (CsNEJs) migrate along the bile duct via bile chemotaxis. Cholic acid is a major component of bile that induces this migration. We investigated the neuronal control of chemotactic behavior of CsNEJs toward cholic acid. The migration of CsNEJs was strongly inhibited at sub-micromolar concentration by dopamine D_1_ (LE-300 and SKF-83566), D_2_ (spiramide, nemonapride, and sulpiride), and D_3_ (GR-103691 and NGB-2904) receptor antagonists, as well as a dopamine reuptake inhibitor (BTCP). Neuropeptides, FMRFamide, peptide YY, and neuropeptide Y were also potent inhibitors of chemotaxis. Meanwhile, serotonergic, glutamatergic, and cholinergic inhibitors did not affect chemotaxis, with the exception of fluoxetine and CNQX. Confocal immunofluorescence analysis indicated that dopaminergic and cholinergic neurons were colocalized in the somatic muscle tissues of adult *C*. *sinensis*. Our findings suggest that dopaminergic neurons and neuropeptides play a major role in the chemotactic migration of CsNEJs to bile, and their inhibitors or modulators could be utilized to prevent their migration from the bile duct.

## Author summary

The liver fluke *Clonorchis sinensis* is a flatworm parasite dwelling in the bile duct, which can induce serious pathological inflammatory changes, and chronic infection is associated with bile duct cancer. In order to gain access to its habitat, *C*. *sinensis* larva follows chemical cues from the liver, a phenomenon called chemotaxis. Bile, including its component cholic acid, is essential for the juvenile fluke’s migration toward the intrahepatic bile duct from the host intestine. Here, we report that the chemotaxis to cholic acid is controlled through dopaminergic neurons and neuropeptides. This observation can be utilized to develop a preventive intervention against infection by *C*. *sinensis*.

## Introduction

*Clonorchis sinensis* is the most common human liver fluke in East Asia, with more than 200 million people at risk of infection [1]. It primarily inhabits the bile duct, where it can induce serious pathological inflammatory changes, and chronic infection is associated with the development of cholangiocarcinoma [2]. Infections occur via the consumption of raw freshwater fish carrying *C*. *sinensis* metacercariae. The ingested metacercariae excyst in the duodenum, and the newly excysted juveniles (hereafter termed CsNEJs) quickly migrate to the intrahepatic bile duct in response to chemotactic cues in the bile [3,4].

In *C*. *sinensis* metacercariae, bile stimulates the expression of genes for energy generation to induce migration and those associated with maturation [5]. The presence of a bile acid transporter also indicates the role of bile for their survival and migration [6]. Indeed, cholic acid in bile has been found to be a primary attractant for CsNEJs to migrate into the intrahepatic bile duct [7]. The nervous system governing the cholic acid-induced chemotactic movement is, however, completely unknown.

Helminthic locomotion, invasion and attachment are related to the neuromuscular system [8–12]. Neurotransmitters such as dopamine, serotonin, glutamate, acetylcholine, and neuropeptides have been observed to control the behavior. However, these processes are not well described in *C*. *sinensis*. In this study, we used a pharmacological approach to elucidate the neuronal control over the chemotaxis of CsNEJs to cholic acid.

## Materials and methods

### Ethics statement

A New Zealand White rabbit (2.2 kg) was purchased from Samtako Bio Korea Inc. (Osan, South Korea). Animal was handled in an accredited Chung-Ang University animal facility in accordance with the AAALAC (Association for Assessment and Accreditation of Laboratory Animal Care) International Animal Care policies (Accredited Unit, Korea FDA; Unit Number 36). Approval for animal experiments was obtained from the Institutional Review Board of Chung-Ang University animal facility (Approval Number CAU-2011-0053).

### Preparation of *C*. *sinensis* newly excysted juveniles

Topmouth gudgeon (*Pseudorasbora parva*), the second intermediate host of *C*. *sinensis*, were purchased at a fish market in Shenyang, Liaoning Province, People’s Republic of China. Fish were ground and digested in artificial gastric fluid (0.5% pepsin, pH 2.0, MP Biochemicals Co., Solon, OH) for 2 h at 37°C [13]. Solid matter was removed from digested content by filtration through a sieve of 212 μm mesh diameter. *C*. *sinensis* metacercariae were collected using sieves of 106 and 53 μm mesh diameter and washed with 0.85% saline, then collected under a dissecting microscope and stored in phosphate-buffered saline (PBS) at 4°C until use. The metacercariae were excysted in 0.005% trypsin (Difco, Sparks, MD) and used as experimental CsNEJs for downstream assays.

### Inhibition assay for bile-chemotactic migration

A multi-trough bile chemotaxis assay panel was used per a previous study’s design [7]. Eight semi-cylindrical troughs 10 cm long, 1 cm wide, and 0.5 cm deep were carved into a polycarbonate block. Each trough was graduated 0 at the center, +1 to +5 cm on the left side, and −1 to −5 cm on the right side.

In all chemotaxis assays, each trough was filled with 1 mL of 1× Locke’s buffer as a base solution [3] with various concentrations of the test compounds. Approximately twenty CsNEJs were placed at the center 0 point of each trough using a micropipette. After acclimating for 10 min, CsNEJs were attracted by dropping 4 μL of 50 mM cholic acid (Sigma-Aldrich Co., St. Louis, MO) dissolved in dimethyl sulfoxide (DMSO, Sigma-Aldrich) at the +5 point. The same volume of DMSO only was dropped at the +5 point as a negative control. Behavior and migration distance of CsNEJs were observed using a dissecting microscope every 10 min for 60 min. All assays were performed inside a walk-in chamber maintained at 37°C and 80% humidity. To minimize temperature fluctuation, the chemotaxis panel was covered with a lid except when chemicals were applied or CsNEJs were observed.

Effects of neuro-antagonists on the chemotaxis of CsNEJs were measured. Dopaminergic inhibitors used were LE-300, SKF-83566, sulpiride, remoxipride, nemonapride, spiramide (AMI-193), NGB-2904, U-99194, GR-103691, and benzothiophenylcyclohexylpiperidine (BTCP). Serotoninergic inhibitors were fluoxetine, spiroxatrine, ritanserin, and Y-25130. Glutamatergic inhibitors were CNQX (6-cyano-7-nitroquinoxaline-2,3-dione), NBQX (6-nitro-2,3-dioxo-1,2,3,4-tetrahydrobenzo[f]quinoxaline-7-sulfonamide), MK-801, and cyclothiazide. Cholinergic inhibitors were pirenzepine and benzoquinonium. Neuropeptides were Phe-Met-Arg-Phe amide (FMRFamide), neuropeptide Y (NPY), and peptide YY (PYY).

All test compounds (Sigma-Aldrich) were dissolved in 99.5% ethanol at stock concentration of 10 mM. Stock solution was either made on the day of assay or taken from aliquots stored at −20°C for no longer than 4 weeks prior to use. Each stock solution was serially diluted in 1× Locke’s solution.

### Data analysis

Mean chemotactic distance (mm) was calculated as the migration distance of all CsNEJs and dividing by their number. Percent chemotactic inhibition was calculated as a ratio of migration distance difference between test and positive control groups. All assays were performed in triplicate with different batches of CsNEJs and results presented as a mean ± standard error of the mean. Difference was statistically analyzed using Student’s *t*-test and considered statistically significant at *P* < 0.05.

### Confocal microscopy

Approximately 300 *C*. *sinensis* metacercariae were fed to a New Zealand White rabbit. Adult *C*. *sinensis* were recovered from the bile duct of the rabbit 4 months post-infection, then flat-fixed in 4% paraformaldehyde in 0.1 M PBS for 1 h, washed three times with AbD solution (0.1 M PBS, 0.1% Triton X-100, 1% bovine serum albumin, and 0.1% NaN_3_, at pH 7.4) for 10 min each, and incubated in AbD solution for 24 h at 4°C. The flukes were incubated in primary antibody solution containing goat anti-choline acetyltransferase polyclonal antibody (1:10 diluted, Millipore, Billerica, MA) and mouse anti-tyrosine hydroxylase monoclonal antibody (1:250 diluted, Millipore) for 5 d at 4°C. After washing in AbD for 24 h at 4°C, the flukes were incubated in donkey-anti-goat-Cy3 and donkey-anti-mouse-FITC secondary antibodies (1:500 diluted) for 5 d at 4°C. After a final overnight wash in AbD, the immune-stained flukes were mounted in Gel/Mount (Biomeda, Foster City, CA). The specimens were observed and photographed under a confocal microscope.

## Results

### Effects of dopaminergic antagonists on chemotaxis of CsNEJs toward cholic acid

**Fig 1:**
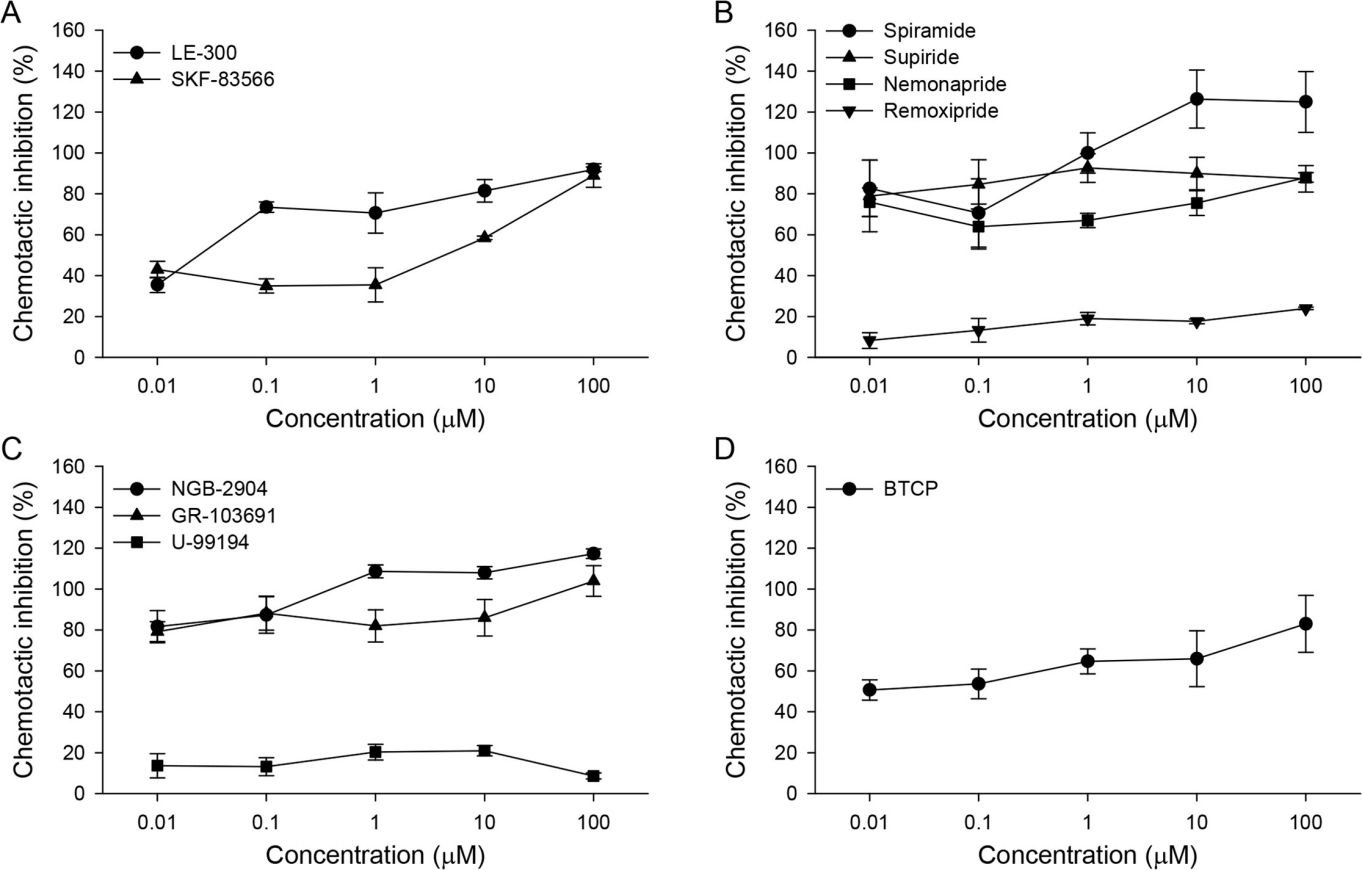
Inhibition of chemotactic migration of CsNEJs to cholic acid by dopaminergic inhibitors. Percent inhibition of chemotactic migration of CsNEJs toward 50 mM cholic acid by dopamine D_1_ (A), D_2_ (B), and D_3_ (C) receptor antagonists, and a dopamine reuptake inhibitor (D). CsNEJs were incubated in a solution containing a test compound at 0.01–100 μM for 10 min, after which 50 mM cholic acid was placed at the positive end to induce chemotaxis. Migration distance of CsNEJs over 60 min in the presence of each compound was subtracted from the control migration distance, and the ratio is presented as percent inhibition.

Neuronal control of CsNEJs’ chemotaxis toward cholic acid was investigated. Various pharmacological agents acting on neuroreceptors such as dopamine, glutamate, serotonin, acetylcholine, and neuropeptide receptors were tested (Table 1). Of these, dopaminergic antagonists noticeably inhibited chemotactic migration of CsNEJs to 50 mM cholic acid, even at nanomolar concentrations (Fig 1). Inhibition showed some degree of concentration dependency.

**Table 1:** List of neurotransmitter inhibitors and neuropeptides.

Group	Compound	Receptor specificity
Dopamine	LE-300	D_1_ receptor antagonist
	SKF-83566	D_1_ receptor antagonist
	Sulpiride	D_2_ and D_3_ receptor antagonist
	Remoxipride	D_2_ and D_3_ receptor antagonist
	Nemonapride	D_2_ and D_3_ receptor antagonist
	Spiramide (AMI-193)	D_2_, 5-HT_2A_ and 5-HT_1A_ receptor antagonist
	NGB-2904	D_3_ receptor antagonist
	U-99194	D_3_ receptor antagonist
	GR-103691	D_3_ receptor antagonist
	BTCP	Dopamine reuptake inhibitor
Serotonin	Fluoxetine	Selective serotonin reuptake inhibitor
	Spiroxatrine	5-HT_1A_ receptor antagonist
	Ritanserin	5-HT_2A_ and 5-HT_2C_ receptor antagonist
	Y-25130	5-HT_3_ receptor antagonist
Glutamate	CNQX	AMPA/kainate receptor antagonist
	NBQX	AMPA receptor antagonist
	MK-801	NMDA receptor antagonist
	Cyclothiazide	Positive AMPA/kainate receptor modulator
Acetylcholine	Pirenzepine	Muscarinic M_1_ receptor antagonist
	Benzoquinonium	Nicotinic receptor antagonist
Neuropeptide	FMRFamide	FMRFaR receptor agonist
	Neuropeptide Y (NPY)	Y_1-5_ receptor agonist
	Peptide YY (PYY)	Y_2_ receptor agonist

Dopamine D_1_ receptor antagonists LE-300 and SKF-83566 as low as 10 nM inhibited chemotaxis of CsNEJs toward 50 mM cholic acid by 35.5 ± 3.8% and 43.0 ± 4.0%, respectively (Fig 1A). This inhibition increased with concentration of antagonists, and LE-300 and SKF-83566 at 100 μM inhibited chemotaxis as much as 92.0 ± 1.2% and 89.0 ± 5.8%.

Dopamine D_2_ receptor antagonists spiramide, sulpiride, and nemonapride inhibited chemotaxis even more strongly (Fig 1B), and remoxipride did so to a lesser degree. Spiramide, sulpiride, nemonapride, and remoxipride at 10 nM inhibited the chemotaxis by 82.7 ± 13.9%, 79.0 ± 17.6%, 76.0 ± 6.9%, and 8.3 ± 3.8%, respectively, and at 100 μM by 125 ± 14.9%, 87.3 ± 6.4%, 88.0 ± 2.3%, and 24.0 ± 0.6%. Peculiarly, CsNEJs moved in the opposite direction from cholic acid in the presence of 10–100 μM spiramide.

Dopamine D_3_ receptor antagonists NGB-2904 and GR-103691 were also potent inhibitors of chemotaxis. U-99194 was only a weak inhibitor (Fig 1C). At 10 nM, NGB-2904 and GR-103691 inhibited chemotaxis by 81.7 ± 7.9% and 79.3 ± 4.9%, and at 100 μM by 117.3 ± 2.3% and 104.0 ± 7.5%, respectively. Both NGB-2904 and GR-103691 caused CsNEJs to avoid cholic acid at high micromolar concentrations.

A dopamine reuptake inhibitor BTCP moderately inhibited chemotaxis (Fig 1D). At 10 nM, BTCP inhibited chemotaxis by 50.7 ± 5.0%, increasing to 83.0 ± 13.9% at 100 μM.

Although dopaminergic inhibitors suppressed chemotaxis to cholic acid, they did not decrease motility or cause shrinkage of the CsNEJs.

### Effects of serotonergic, glutamatergic, and cholinergic inhibitors on chemotaxis of CsNEJs toward cholic acid

**Fig 2:**
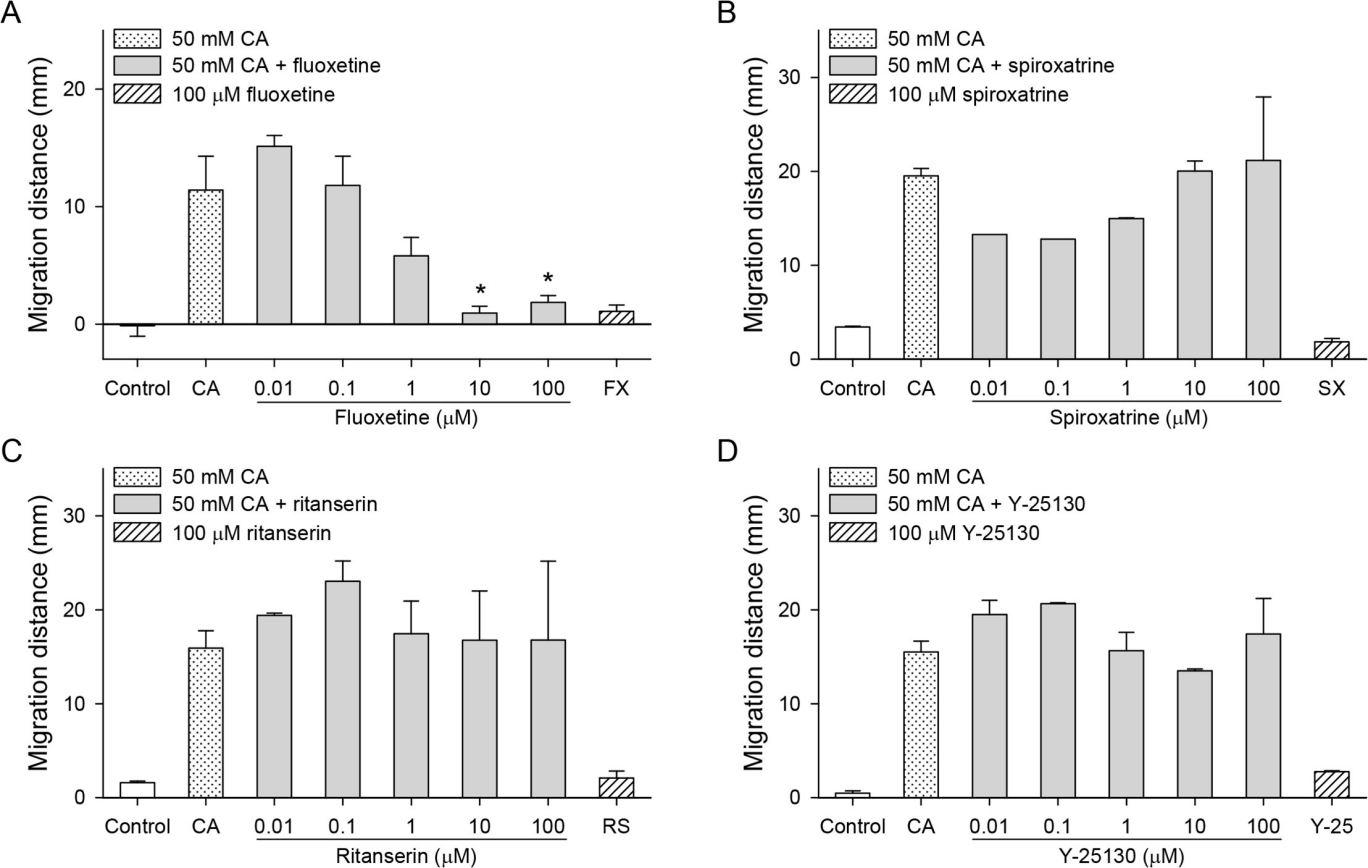
Effects of serotonergic inhibitors on the cholic acid-induced chemotaxis of CsNEJs. CsNEJs were stimulated with 50 mM cholic acid (CA) in the presence of fluoxetine (A), spiroxatrine (B), ritanserin (C), or Y-25130 (D), and migration distance was measured at 60 min. Asterisk * indicates *P* < 0.05 compared to 50 mM cholic acid only.

Serotonergic antagonists such as spiroxatrine (5-HT_1_), ritanserin (5-HT_2_), and Y-25130 (5-HT_3_) up to 100 μM did not influence the chemotactic migration of CsNEJs toward cholic acid (Fig 2B–2D). However, fluoxetine, a selective serotonin reuptake inhibitor, reduced chemotaxis at concentrations higher than 1 μM (Fig 2A, *P* < 0.05). Fluoxetine alone at 100 μM caused a shrinkage of the worms.

**Fig 3:**
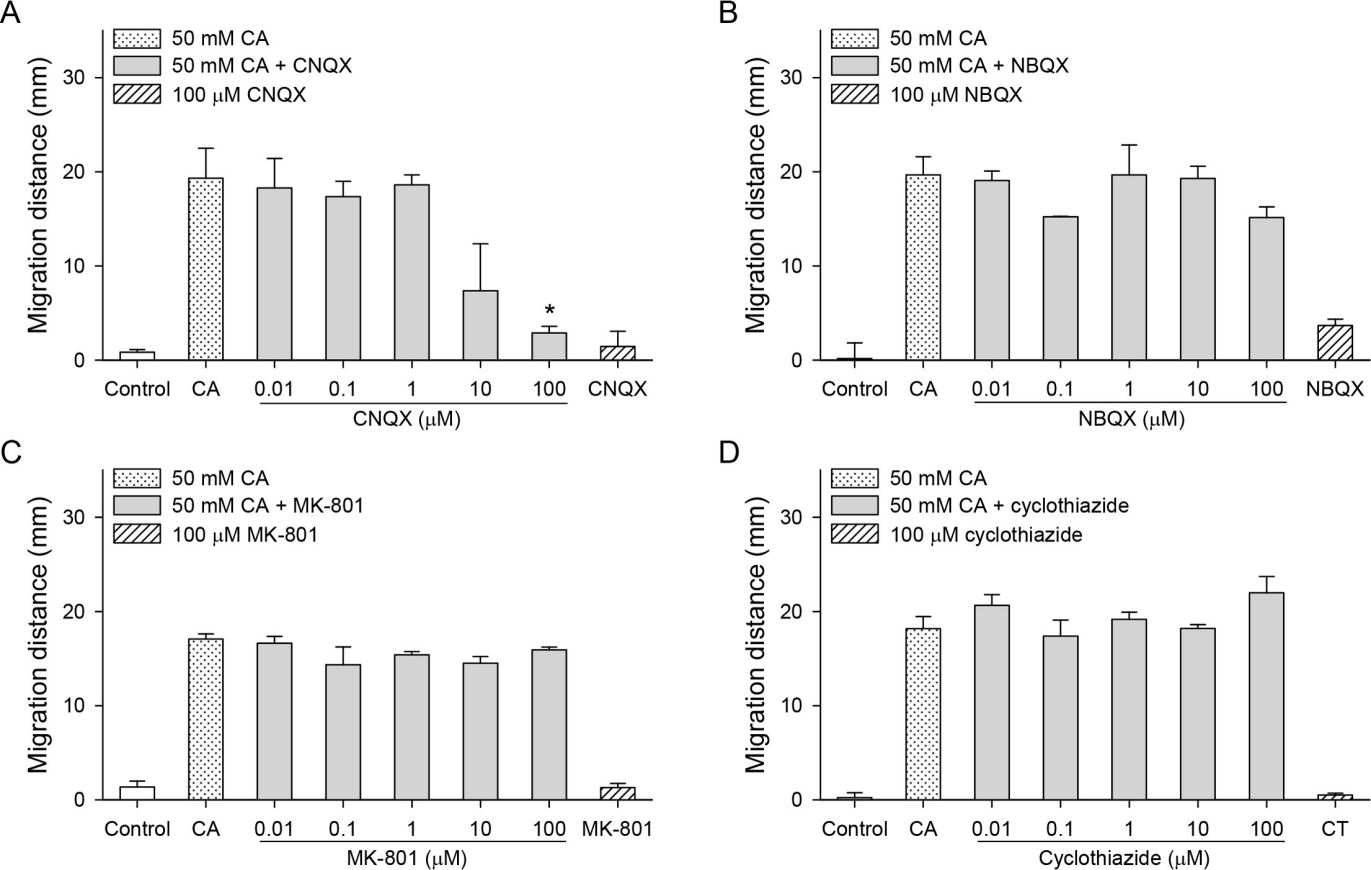
Effects of glutamatergic inhibitors on the cholic acid-induced chemotaxis of CsNEJs. CsNEJs were stimulated with 50 mM cholic acid (CA) in the presence of CNQX (A), NBQX (B), MK-801 (C), or cyclothiazide (D), and migration distance was measured at 60 min. Asterisk * indicates *P* < 0.05 compared to 50 mM cholic acid only.

A glutamate AMPA (α-amino-3-hydroxy-5-methyl-4-isoxazole-propionic acid)/kainate receptor antagonist CNQX inhibited chemotaxis only at a high concentration of 100 μM (Fig 3A, *P* < 0.05). However, the AMPA receptor antagonist NBQX, the NMDA (*N*-methyl-d-aspartate) receptor antagonist MK-801, and the positive AMPA/kainate receptor modulator cyclothiazide did not inhibit chemotaxis (Fig 3B–3D).

**Fig 4:**
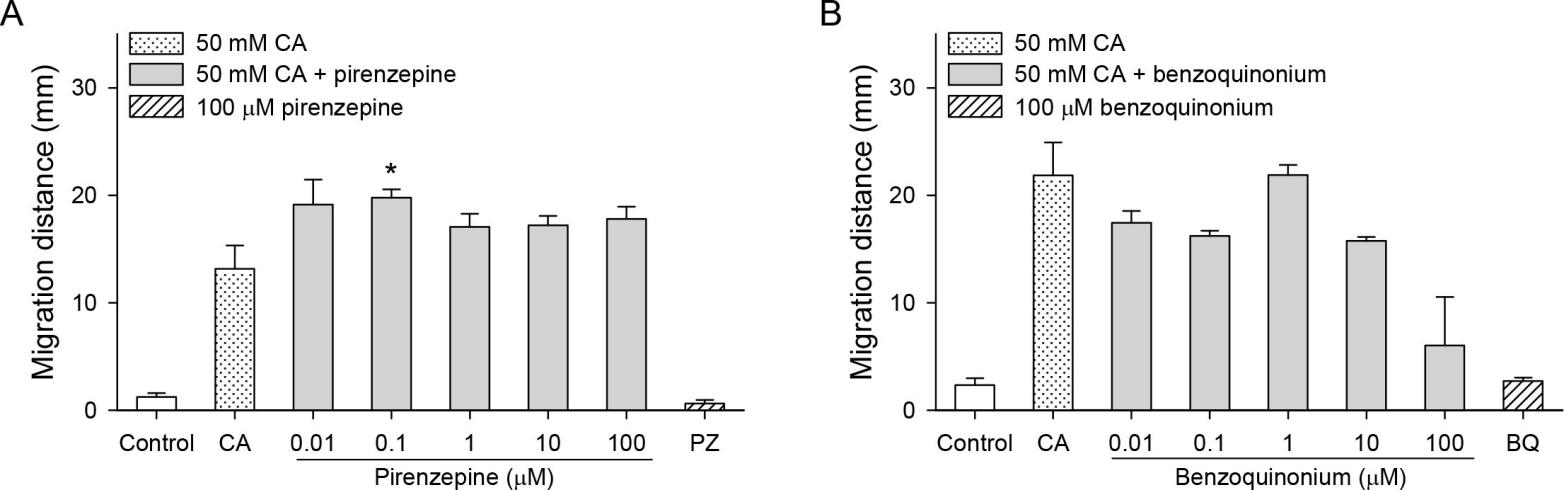
Effects of cholinergic inhibitors on the cholic acid-induced chemotaxis of CsNEJs. CsNEJs were stimulated with 50 mM cholic acid (CA) in the presence of pirenzepine (A) or benzoquinonium (B), and migration distance was measured at 60 min. Asterisk * indicates *P* < 0.05 compared to 50 mM cholic acid only.

Pirenzepine, a muscarinic receptor antagonist, and benzoquinonium, a nicotinic receptor antagonist, did not inhibit chemotaxis (Fig 4), and pirenzepine at 0.1 μM actually enhanced it (*P* < 0.05).

### Effects of neuropeptides on chemotaxis of CsNEJs toward cholic acid

**Fig 5:**
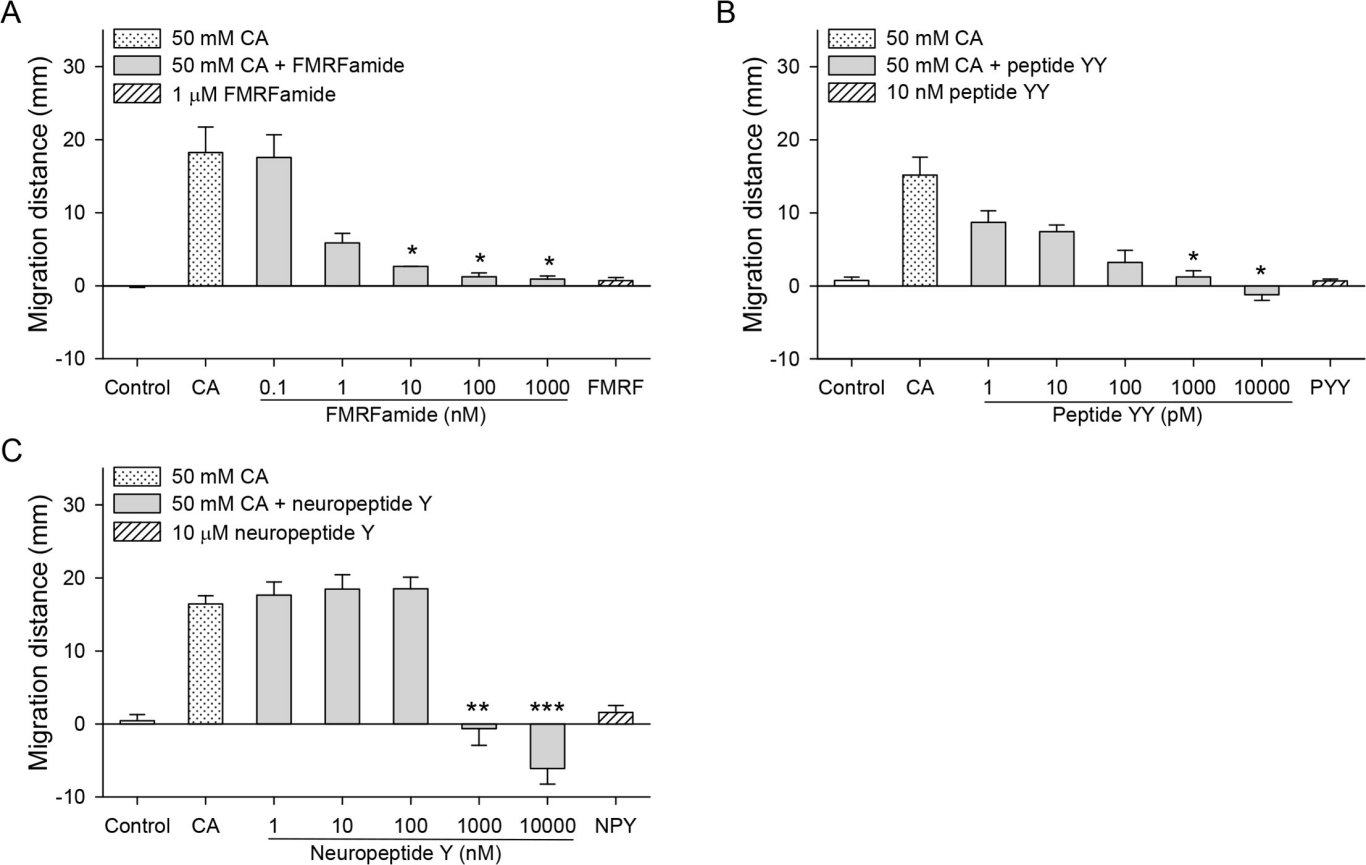
Effects of neuropeptides on the cholic acid-induced chemotaxis of CsNEJs. CsNEJs were stimulated with 50 mM cholic acid (CA) in the presence of FMRFamide (A), peptide YY (B), or neuropeptide Y (C), and migration distance was measured at 60 min. Asterisks indicate * *P* < 0.05, ** *P* < 0.01, and *** *P* < 0.001 compared to 50 mM cholic acid only.

Neuropeptides such as FMRFamide, peptide YY, and neuropeptide Y strongly inhibited chemotactic migration of CsNEJs at sub-micromolar to low micromolar concentrations (Fig 5), where FMRFamide at 10 nM inhibited chemotaxis by 85%, and peptide YY at 1 nM by 92% (Fig 5A and 5B, *P* < 0.05). The inhibition by these neuropeptides showed concentration dependency. On the other hand, neuropeptide Y did not affect chemotaxis up to 100 nM, but at over 1 μM, CsNEJs suddenly avoided CA, in an all-or-none manner (Fig 5C). All three neuropeptides when applied alone had no effect on direction of migration.

### Immunolocalization of dopaminergic and cholinergic neurons in adult *C*. *sinensis*

**Fig 6:**
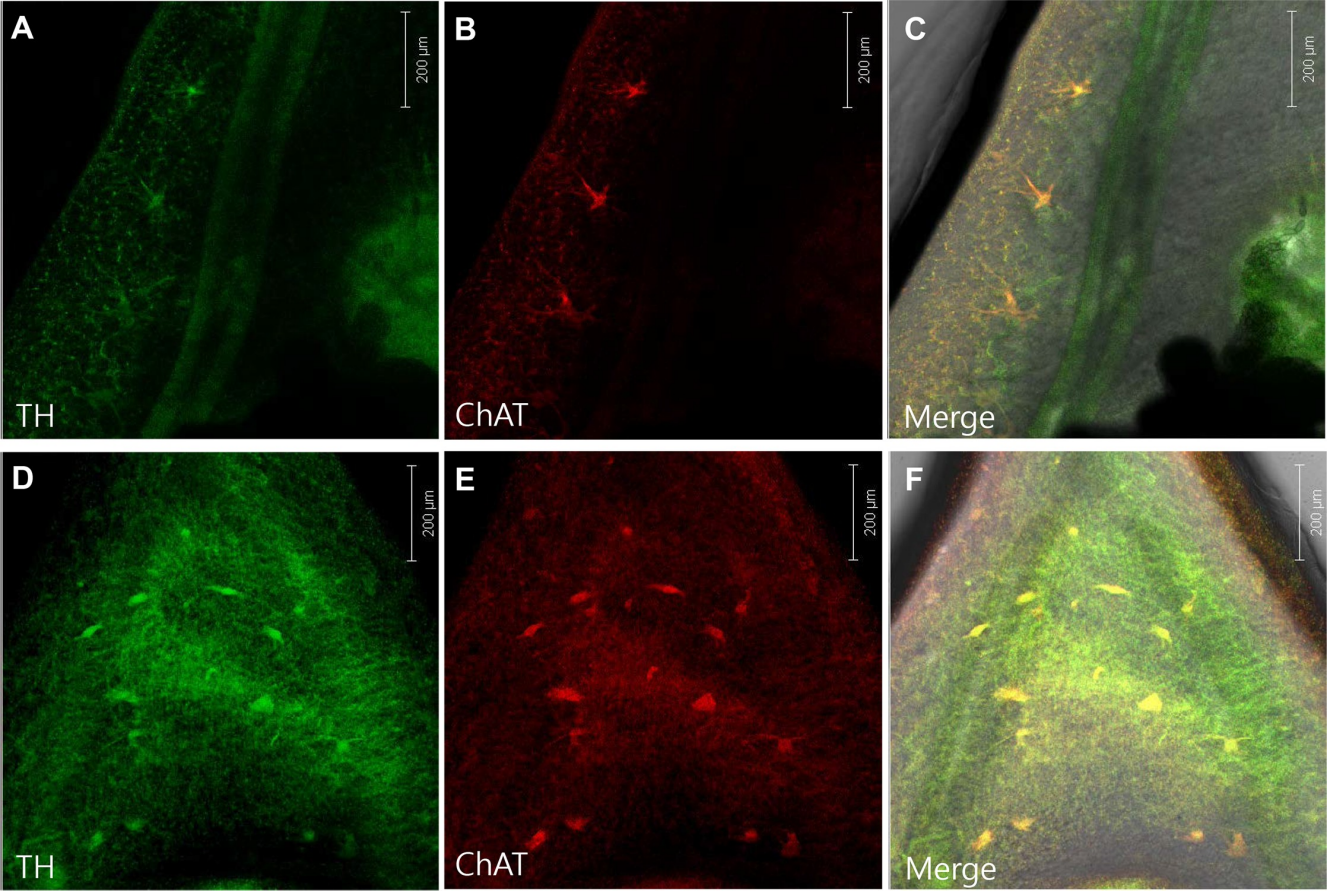
Confocal immunofluorescent micrographs of adult *C*. *sinensis*. Double-immunostaining with antibodies to tyrosine hydroxylase (TH) (A, D), choline acetyltransferase (ChAT) (B, E), and images merged (C, F). Upper panels show the lateral margin to the level of the ventral sucker, and lower panels show the area between the esophagus and ventral sucker.

Confocal immunofluorescence microscopy indicated tissue distribution of dopaminergic and cholinergic neurons in adult *C*. *sinensis*. Tyrosine hydroxylase (TH) and choline acetyltransferase (ChAT) antibodies were used to detect dopaminergic and cholinergic neurons, respectively. Anti-TH green fluorescence and anti-ChAT red fluorescence indicated the colocalized presence of both neuronal types at low density throughout the body of adult *C*. *sinensis*, appearing more frequently in regions between oral and ventral suckers in the forebody, and between the testes in the hindbody. The neurons were aligned in the lateral margin of the intestine in the forebody (Fig 6A–6F). The oral and ventral suckers were satellited with these neuronal cell bodies.

## Discussion

The central nervous system of *C*. *sinensis* is composed of two cerebral ganglia whose anterior nerves contribute to the pharynx and to the oral sucker [14]. Serotonin has been found to stimulate the motility of adult *C*. *sinensis*. However, cholinergic agonists have an opposite effect, which is not reversed by traditional cholinergic antagonists, suggesting a disparate pharmacological profile of helminthic acetylcholine receptors from mammalian counterparts, or a non-receptor mediated action [15]. Nevertheless, the anthelmintic drug tribendimidine is a nicotinic acetylcholine receptor agonist effective to treat *C*. *sinensis* infection [16]. In the present study, however, the nicotinic antagonist benzoquinonium tended to inhibit chemotaxis of CsNEJs to cholic acid, whereas a muscarinic antagonist pirenzepine showed a tendency to enhance it.

Dopamine neurons play a major role in the movement and muscle function of other flatworm and roundworm species [9,17–22]. The existence of a dopamine neuron, however, has not been previously established in *C*. *sinensis*. Our immunofluorescence findings confirm its existence in adult *C*. *sinensis*. The neuronal cell bodies were located in the somatic muscle region, suggesting dopaminergic control of its locomotory behavior. In accordance with this, the chemotactic migration of CsNEJs to cholic acid was suppressed by dopamine receptor antagonists even at low nanomolar concentration. Dopamine D_2_ and D_3_ receptor antagonists such as spiramide, sulpiride, nemonapride, NGB-2904, and GR-103691 were particularly powerful inhibitors. These results suggest that dopamine neurons may control the chemotactic migration of CsNEJs to cholic acid. However, since remoxipride and U-99194 were only weakly effective, these results should be regarded with some caution. In addition, it is not certain whether the antagonistic effects on chemotaxis were due to an inhibition of muscle movement or a disturbance of chemosensation.

Curiously, the dopamine reuptake inhibitor BTCP also moderately inhibited chemotaxis. Similarly, anomalous pharmacology has also been observed in the *Schistosoma mansoni* dopamine D_2_ receptor, where both the traditional agonists and antagonists inhibited the receptor [9]. In addition, atropine, a traditional muscarinic acetylcholine receptor antagonist, showed inverse agonist activity toward an acetylcholine receptor [23]. This discrepancy may arise from differences in the molecular structure of receptors between invertebrates and vertebrates.

Serotonin stimulates motility in various flatworms, including adult *C*. *sinensis* [15]. Serotonin receptor is involved in the control of muscle motility at the neuromuscular junction of *Fasciola hepatica* and *S*. *mansoni* [24–26]. Fluoxetine and exogenous serotonin both produce a strongly hyperactive phenotype in *S*. *mansoni* schistosomula [27]. Serotonin also markedly stimulates *F*. *hepatica* activity, whereas fluoxetine oddly inhibits it [22]. We also observed that fluoxetine at a micromolar concentration inhibited chemotaxis, and that fluoxetine alone at 100 μM caused a shrinkage of CsNEJs, possibly indicating a toxic influence unrelated to its inhibition of serotonin transport. Serotonin antagonists inhibit serotonin-induced increases in motility of *S*. *mansoni* sporocyst [28]. However, in the present study, the serotonin antagonists spiroxatrine, ritanserin, and Y-25130 had no noticeable effects on the chemotaxis of CsNEJs. This also may be attributable to species differences in receptor structure in invertebrates.

Glutamate-like immunoreactivity is widespread in the nervous system of *F*. *hepatica* [29], where G protein-coupled glutamate receptors are predicted to exist [25]. Kainate binding sites were observed in adult *S*. *mansoni*, indicating an existence of ionotropic glutamate receptors [30]. However, MK-801, a traditional NMDA antagonist in vertebrates, binds to nicotinic receptors, but not NMDA receptors in adult *S*. *mansoni* [31]. In the present study, only CNQX at 100 μM inhibited chemotaxis of CsNEJs, whereas NBQX, MK-801, and cyclothiazide had no effect. These findings indicate that ionotropic glutamate receptors do not modulate the chemotactic movement of CsNEJs.

Neuropeptides such as FMRFamide-like peptides and neuropeptide Fs are widely distributed in nervous systems in flatworms, including neurons serving the somatic musculature [11,32–34]. G protein-coupled receptors for neuropeptide F/Y binding are predicted to exist in *F*. *hepatica* [25]. FMRFamide, peptide YY, and neuropeptide Y, the neuropeptides tested here, all strongly inhibited the chemotaxis of CsNEJs to cholic acid. In *S*. *mansoni*, FMRFamide-like peptides elicit potent muscle contraction by enhancing Ca^2+^ influx through sarcolemmal voltage-gated Ca^2+^ channels [35]. The potent nature of muscle contraction by neuropeptides may lead to tetanic paralysis of CsNEJs, hampering their chemotactic movement.

In summary, our results represent the first immunohistochemical demonstration of dopaminergic neurons in the somatic muscle layer of adult *C*. *sinensis*, and that dopamine receptor antagonists and neuropeptides are powerful inhibitors of CsNEJs chemotaxis to cholic acid. Since cholic acid is the most important chemoattractant to CsNEJs in bile fluids, this finding could be utilized for the development of drugs preventing *C*. *sinensis* migration to the bile duct [4,7].

## References

[1] Hong ST, Fang Y. *Clonorchis sinensis* and clonorchiasis, an update. Parasitol Int. 2012; 61(1):17–24. 10.1016/j.parint.2011.06.007 21741496

[2] Shin HR, Oh JK, Masuyer E, Curado MP, Bouvard V, Fang YY, et al Epidemiology of cholangiocarcinoma: an update focusing on risk factors. Cancer Sci. 2010; 101(3):579–85. 10.1111/j.1349-7006.2009.01458.x 20085587 PMC11158235

[3] Li S, Kim TI, Yoo WG, Cho PY, Kim TS, Hong SJ. Bile components and amino acids affect survival of the newly excysted juvenile *Clonorchis sinensis* in maintaining media. Parasitol Res. 2008; 103(5):1019–24. 10.1007/s00436-008-1084-3 18587668

[4] Kim TI, Yoo WG, Kwak BK, Seok JW, Hong SJ. Tracing of the bile-chemotactic migration of juvenile *Clonorchis sinensis* in rabbits by PET-CT. PLoS Negl Trop Dis. 2011; 5(12):e1414 10.1371/journal.pntd.0001414 22180795 PMC3236719

[5] Kim TI, Cho PY, Yoo WG, Li S, Hong SJ. Bile-induced genes in *Clonorchis sinensis* metacercariae. Parasitol Res. 2008; 103(6):1377–82. 10.1007/s00436-008-1144-8 18682984

[6] Lu Y, Yoo WG, Dai F, Lee JY, Pak JH, Sohn WM, et al Characterization of a novel organic solute transporter homologue from *Clonorchis sinensis*. PLoS Negl Trop Dis. 2018; 12(4):e0006459 10.1371/journal.pntd.0006459 29702646 PMC5942847

[7] Li S, Yoo WG, Song JH, Kim TI, Hong SJ. Bile acids drive chemotaxis of *Clonorchis sinensis* juveniles to the bile duct. PLoS Negl Trop Dis. 2018; 12(10):e0006818 10.1371/journal.pntd.0006818 30273341 PMC6181427

[8] Sawin ER, Ranganathan R, Horvitz HR. *C*. *elegans* locomotory rate is modulated by the environment through a dopaminergic pathway and by experience through a serotonergic pathway. Neuron. 2000; 26(3):619–31. 10896158 10.1016/s0896-6273(00)81199-x

[9] Taman A, Ribeiro P. Investigation of a dopamine receptor in *Schistosoma mansoni*: functional studies and immunolocalization. Mol Biochem Parasitol. 2009; 168(1):24–33. 10.1016/j.molbiopara.2009.06.003 19545592

[10] Ribeiro P, El-Shehabi F, Patocka N. Classical transmitters and their receptors in flatworms. Parasitology. 2005; 131 Suppl:S19–40. 16569290 10.1017/S0031182005008565

[11] McVeigh P, Kimber MJ, Novozhilova E, Day TA. Neuropeptide signalling systems in flatworms. Parasitology. 2005; 131 Suppl:S41–55. 16569292 10.1017/S0031182005008851

[12] Pax RA, Siefker C, Bennett JL. *Schistosoma mansoni*: differences in acetylcholine, dopamine, and serotonin control of circular and longitudinal parasite muscles. Exp Parasitol. 1984; 58(3):314–24. 6500002 10.1016/0014-4894(84)90048-1

[13] Hong SJ, Seong KY, Sohn WM, Song KY. Molecular cloning and immunological characterization of phosphoglycerate kinase from *Clonorchis sinensis*. Mol Biochem Parasitol. 2000; 108(2):207–16. 10838223 10.1016/s0166-6851(00)00220-6

[14] He YX. Nervous system of *Clonorchis sinensis* as revealed by acetylcholinesterase activity. Southeast Asian J Trop Med Public Health. 1991; 22(3):412–6. 1818393

[15] Shyu LY, Terada M, Lee HH. *In vitro* effects of various neuropharmacological agents on the motility of adult *Clonorchis sinensis*. Kaohsiung J Med Sci. 1998; 14(8):473–9. 9780596

[16] Xiao SH, Utzinger J, Tanner M, Keiser J, Xue J. Advances with the Chinese anthelminthic drug tribendimidine in clinical trials and laboratory investigations. Acta Trop. 2013; 126(2):115–26. 10.1016/j.actatropica.2013.01.009 23352956

[17] Nishimura K, Kitamura Y, Inoue T, Umesono Y, Sano S, Yoshimoto K, et al Reconstruction of dopaminergic neural network and locomotion function in planarian regenerates. Dev Neurobiol. 2007; 67(8):1059–78. 10.1002/dneu.20377 17565705

[18] Jorgensen EM. Dopamine: should I stay or should I go now? Nat Neurosci. 2004; 7(10):1019–21. 10.1038/nn1004-1019 15452567

[19] Ezak MJ, Ferkey DM. The *C*. *elegans* D2-like dopamine receptor DOP-3 decreases behavioral sensitivity to the olfactory stimulus 1-octanol. PLoS One. 2010; 5(3):e9487 10.1371/journal.pone.0009487 20209143 PMC2830454

[20] Hamdan FF, Ribeiro P. Cloning and characterization of a novel form of tyrosine hydroxylase from the human parasite, *Schistosoma mansoni*. J Neurochem. 1998; 71(4):1369–80. 10.1046/j.1471-4159.1998.71041369.x 9751167

[21] Hu Y, Shi D, Luo Q, Liu Q, Zhou Y, Liu L, et al Cloning and characterization of a novel enzyme: tyrosine hydroxylase from *Schistosoma japonicum*. Parasitol Res. 2011; 109(4):1065–74. 10.1007/s00436-011-2347-y 21556690

[22] Holmes SD, Fairweather I. *Fasciola hepatica*: the effects of neuropharmacological agents upon *in vitro* motility. Exp Parasitol. 1984; 58(2):194–208. 6148259 10.1016/0014-4894(84)90035-3

[23] MacDonald K, Kimber MJ, Day TA, Ribeiro P. A constitutively active G protein-coupled acetylcholine receptor regulates motility of larval *Schistosoma mansoni*. Mol Biochem Parasitol. 2015; 202(1):29–37. 10.1016/j.molbiopara.2015.09.001 26365538 PMC4607267

[24] Tembe EA, Holden-Dye L, Smith SW, Jacques PA, Walker RJ. Pharmacological profile of the 5-hydroxytryptamine receptor of *Fasciola hepatica* body wall muscle. Parasitology. 1993; 106 (Pt 1):67–73. 8479803 10.1017/s0031182000074837

[25] McVeigh P, McCammick E, McCusker P, Wells D, Hodgkinson J, Paterson S, et al Profiling G protein-coupled receptors of *Fasciola hepatica* identifies orphan rhodopsins unique to phylum Platyhelminthes. Int J Parasitol Drugs Drug Resist. 2018; 8(1):87–103. 10.1016/j.ijpddr.2018.01.001 29474932 PMC6114109

[26] Patocka N, Sharma N, Rashid M, Ribeiro P. Serotonin signaling in *Schistosoma mansoni*: a serotonin-activated G protein-coupled receptor controls parasite movement. PLoS Pathog. 2014; 10(1):e1003878 10.1371/journal.ppat.1003878 24453972 PMC3894222

[27] Patocka N, Ribeiro P. The functional role of a serotonin transporter in *Schistosoma mansoni* elucidated through immunolocalization and RNA interference (RNAi). Mol Biochem Parasitol. 2013; 187(1):32–42. 10.1016/j.molbiopara.2012.11.008 23246818

[28] Boyle JP, Zaide JV, Yoshino TP. *Schistosoma mansoni*: effects of serotonin and serotonin receptor antagonists on motility and length of primary sporocysts *in vitro*. Exp Parasitol. 2000; 94(4):217–26. 10.1006/expr.2000.4500 10831389

[29] Brownlee DJ, Fairweather I. Immunocytochemical localization of glutamate-like immunoreactivity within the nervous system of the cestode *Mesocestoides corti* and the trematode *Fasciola hepatica*. Parasitol Res. 1996; 82(5):423–7. 8738281 10.1007/s004360050139

[30] Mendonca-Silva DL, Pessoa RF, Noel F. Evidence for the presence of glutamatergic receptors in adult *Schistosoma mansoni*. Biochem Pharmacol. 2002; 64(9):1337–44. 10.1016/s0006-2952(02)01358-8 12392816

[31] Pessoa RF, Castro NG, Noel F. Binding of [^3^H]MK-801 in subcellular fractions of *Schistosoma mansoni*: evidence for interaction with nicotinic receptors. Biochem Pharmacol. 2005; 69(10):1509–16. 10.1016/j.bcp.2005.03.005 15857615

[32] Day TA, Maule AG, Shaw C, Pax RA. Structure-activity relationships of FMRFamide-related peptides contracting *Schistosoma mansoni* muscle. Peptides. 1997; 18(7):917–21. 9357046 10.1016/s0196-9781(97)00073-9

[33] Marks NJ, Maule AG, Halton DW, Geary TG, Shaw C, Thompson DP. Pharmacological effects of nematode FMRFamide-related peptides (FaRPs) on muscle contractility of the trematode, *Fasciola hepatica*. Parasitology. 1997; 114 (Pt 6):531–9. 9172424

[34] Graham MK, Fairweather I, McGeown JG. The effects of FaRPs on the motility of isolated muscle strips from the liver fluke, *Fasciola hepatica*. Parasitology. 1997; 114 (Pt 5):455–65. 9149416 10.1017/s0031182096008712

[35] Novozhilova E, Kimber MJ, Qian H, McVeigh P, Robertson AP, Zamanian M, et al FMRFamide-like peptides (FLPs) enhance voltage-gated calcium currents to elicit muscle contraction in the human parasite *Schistosoma mansoni*. PLoS Negl Trop Dis. 2010; 4(8):e790 10.1371/journal.pntd.0000790 20706630 PMC2919380

